# Desensitization by Progressive Up-Titration Prevents First-Dose Effects on the Heart: Guinea Pig Study with Ponesimod, a Selective S1P_1_ Receptor Modulator

**DOI:** 10.1371/journal.pone.0074285

**Published:** 2013-09-12

**Authors:** Markus Rey, Patrick Hess, Martine Clozel, Stéphane Delahaye, John Gatfield, Oliver Nayler, Beat Steiner

**Affiliations:** Actelion Pharmaceuticals Ltd., Allschwil, Switzerland; Universidade Federal do Rio de Janeiro, Brazil

## Abstract

Ponesimod, a selective S1P_1_ receptor modulator, reduces the blood lymphocyte count in all tested species by preventing egress of T and B cells from thymus and peripheral lymphoid organs. In addition, ponesimod transiently affects heart rate and atrioventricular (AV) conduction in humans, effects not observed in mice, rats, and dogs with selective S1P_1_ receptor modulators, suggesting that the regulation of heart rate and rhythm is species dependent. In the present study, we used conscious guinea pigs implanted with a telemetry device to investigate the effects of single and multiple oral doses of ponesimod on ECG variables, heart rate, and blood pressure. Oral administration of ponesimod did not affect the sinus rate (P rate) but dose-dependently induced AV block type I to III. A single oral dose of 0.1 mg/kg had no effect on ECG variables, while a dose of 3 mg/kg induced AV block type III in all treated guinea pigs. Repeated oral dosing of 1 or 3 mg/kg ponesimod resulted in rapid desensitization, so that the second dose had no or a clearly reduced effect on ECG variables as compared with the first dose. Resensitization of the S1P_1_ receptor in the heart was concentration dependent. After desensitization had been induced by the first dose of ponesimod, the cardiac system remained desensitized as long as the plasma concentration was ≥75 ng/ml. By using a progressive up-titration regimen, the first-dose effect of ponesimod on heart rate and AV conduction was significantly reduced due to desensitization of the S1P_1_ receptor. In summary, conscious guinea pigs implanted with a telemetry device represent a useful model to study first-dose effects of S1P_1_ receptor modulators on heart rate and rhythm. This knowledge was translated to a dosing regimen of ponesimod to be tested in humans to avoid or significantly reduce the first-dose effects.

## Introduction

Ponesimod is a selective sphingosine 1-phosphate receptor 1 (S1P_1_) modulator under investigation for the treatment of multiple sclerosis and psoriasis [1], [2]. Sphingosine 1-phosphate (S1P), a lipid mediator derived from membrane sphingolipids, is involved in a myriad of physiological processes throughout the body, including the immune, cardiovascular, and central nervous systems [3], [4]. The effects of extracellular S1P are mediated by five G protein-coupled receptors (GPCR), S1P_1_ to S1P_5_, which exhibit different patterns of tissue expression and are linked to diverse physiological responses [4]. S1P and the S1P_1_ receptor are essential for lymphocyte trafficking and regulate egress of T and B cells from thymus and peripheral lymphoid organs [5], [6]. Because S1P_1_ modulators such as ponesimod prevent lymphocyte recirculation, they reduce the blood lymphocyte count, and are under investigation for the treatment of lymphocyte-mediated inflammatory and autoimmune diseases. The first drug in this class, the non-selective S1P receptor modulator fingolimod, was approved in 2010 for the treatment of patients with relapsing multiple sclerosis.

In rats, dogs and humans, ponesimod reduced blood lymphocyte count in a dose-dependent manner, and the reduction in lymphocyte count was maintained upon repeated dosing [2], [7]. In healthy human subjects, ponesimod was generally well tolerated, but induced first-dose effects on heart rate and, in some subjects, on AV conduction [7]. In rats and dogs, these cardiac effects upon oral administration of ponesimod were not observed. The ponesimod findings in humans were unexpected because the reduction of the heart rate in rodents had been attributed to S1P_3_ activation [8], [9]. Since fingolimod reduces heart rate on initial dosing in humans as well as in rats [8], [10], whereas the selective S1P_1_ modulator ponesimod induces these effects in humans but not in rats, it became apparent that regulation of heart rate and rhythm in humans is different than in rats, namely regulated by S1P_1_ and not by S1P_3_ receptor activation.

Although the cardiac effects of ponesimod are transient and generally asymptomatic, they necessitate patient monitoring during treatment initiation. Eliminating or reducing these first-dose effects could improve convenience for both patients and health care providers. To better understand the S1P_1_-mediated first-dose effects, a thorough search for a preclinical model that closely mimics the human findings was initiated. Conscious guinea pigs implanted with a telemetry transmitter represent a suitable model for studying electrocardiogram (ECG) variables *in vivo* after oral administration of a test compound [11], [12]. In addition, in contrast to mice and rats the shape of the guinea pig ECG is similar to that of humans, with clearly defined P, Q, R, S and T waves [13].

The present study had the following objectives: First, to study the dose-dependent effects of the selective S1P_1_ modulator ponesimod on ECG variables, heart rate and blood pressure in conscious guinea pigs implanted with a telemetry device. Second, to investigate desensitization of the effects on the ECG following single and repeated oral administration and to establish relationships between dosing interval, plasma concentration and desensitization kinetics. Third, to design and test a progressive up-titration regimen with the aim of eliminating or significantly reducing the first-dose effects of ponesimod on ECG variables.

## Materials and Methods

### Ethics Statement

All of the experimental procedures were conducted in accordance with the Swiss animal welfare ordinance and Actelion Animal Welfare policy on the use of experimental animals. The study was approved by the Baselland Cantonal Veterinary Home Office (license no. 164).

### Animals

Normotensive male Dunkin Hartley guinea pigs (body weight 347 to 450 g) were obtained from Harlan Laboratories (Horst, the Netherlands). Guinea pigs were group housed during the acclimatization period (at least 7 days) and pair-housed 4 days after implantation of the telemetry device, with appropriate environmental enrichment (shelter, hay, straw). All animals were maintained under identical conditions and had free access to drinking water and normal pelleted food (No 3418, Provimi Kliba SA, CH-4303 Kaiseraugst). Animals were housed in climate-controlled conditions (18–22°C and 40–60% humidity) with a 12-h light/dark cycle in accordance with the guidelines of the Basel-Landschaft Cantonal Veterinary Office (license N° 164).

### Telemetry System Implantation and Data Collection

After the acclimatization period, guinea pigs were treated with vitamin C (1 g/l, Fluka GmbH, N° 95210, Buchs, CH) in drinking water for at least 1 week before and 2 weeks after surgery. On the day of surgery, guinea pigs were treated with an antibiotic (enrofloxacin, 10 mg/kg s.c., Baytril 2.5%, Bayer, Provet AG, Lyssach, CH). Immediately before surgery, animals were pretreated with an analgesic, tramadol hydrochloride (20 mg/kg s.c., Tramal, Grünenthal, Mitlödi, CH). Anesthesia was induced and maintained by administration of 2 to 3% isoflurane (70% O_2_+30% N_2_O, Carbagas, Basel, Switzerland) by inhalation. The telemetry device was microsurgically implanted in the peritoneal cavity under sterile conditions. In brief, the blood pressure-sensing catheter was placed in the descending aorta below the renal arteries, pointing upstream, the ECG electrodes were implanted to record a lead II Apex-Base ECG as previously described [12]; adherence to the protocol is essential for successful telemeter implantation in this species.

During recovery from anesthesia, the guinea pigs were treated with vitamin C (60 mg/kg s.c.,Vitamin C 10%, G. Streuli & Co. AG, Uznach, Switzerland) and carprofen (10 mg/kg, Rimadyl, Pfizer AG, Zürich, Switzerland). Vitamin C (60 mg/kg) and tramadol hydrochloride (20 mg/kg) were administered subcutaneously once a day for 3 days after surgery.

Telemetry units were obtained from Data Sciences International (St. Paul, MN, USA). The implantable transmitter (model C50-PXT) was designed to measure arterial blood pressure and ECG intervals. Implants were gas sterilized and provided pre-calibrated (relative to a vacuum) by the manufacturer. Before implantation, transmitter calibration was verified at room temperature to be accurate within 3 mmHg.

Arterial blood pressure (ABP) and ECG data were collected using Dataquest ART Gold (Data Sciences International, St. Paul, MN, USA) acquisition system (version 3.01). ABP signals were sampled at 500 Hz. Systolic arterial pressure, diastolic arterial pressure; mean arterial pressure, and heart rate were collected over 10 s at 1-min intervals during the entire experiment, resulting in a series of 5760 data points per day (24 h×60 min×4 variables) for each guinea pig. Fifteen-minute arterial blood pressure and heart rate means were calculated. Heart rate was derived from the blood pressure waveform. ECG signals were sampled at 1000 Hz. ECG waveforms were collected over 10 s at 1-min time intervals.

### Data Analysis

PR and RR intervals were analyzed using EMKA software (ECG-Auto, version 1.5.8.15, Paris, France). ABP is expressed in millimeters of mercury (mmHg), heart rate in beats per minute (bpm). ECG intervals are expressed in milliseconds (ms).

Atrioventricular (AV) block is defined as a delay or interruption in conduction between the atria and ventricles. The heart blocks are divided into three types or degrees.

First-degree heart block (AV block I) on the surface ECG in conscious guinea pigs was defined as a PR interval of >90 ms, which represents an approximately 10% increase vs the average PR interval in controls. Each P wave is followed by a QRS complex.

Second-degree heart block (AV block II) is characterized by some P waves being blocked at the AV node. This results in P waves occurring without a following QRS complex. There are two different types of AV block II: 1) AV block II, Wenckebach, also called Mobitz I, is characterized by a progressive prolongation of the PR interval before failure of an atrial impulse to be conducted to the ventricles (QRS complex is not generated). After an AV block II, Wenckebach the PR interval immediately returns to the baseline interval and the sequence begins again. 2) An AV block II, Mobitz II is characterized by sudden failure of a P wave to be conducted to the ventricles. The PR interval remains constant. Intermittently, beats are not conducted and QRS complexes are dropped, usually in a repeating cycle of every 2^nd^ (2∶1 block) or 3^rd^ (3∶1 block) P wave.

Third-degree heart block (AV block III), also known as complete heart block, is seen on the surface ECG as completely dissociated P waves and QRS complexes, each firing at their own pacemaker rate. The atrial impulse is never conducted to the ventricles.

For detection of AV block I the PR intervals were measured. For AV blocks II to III a visual analysis of the waveforms was performed. P and R rates were manually calculated for each guinea pig using 10 s ECG waveform printouts at the following time points: −30, 0, 30, 60, 90, 120, 150, and 180 min. Means ± SEM were calculated.

### Effect of Ponesimod on Heart Rate and ECG Intervals

Ponesimod or its vehicle (methylcellulose 0.25% containing 0.05% Tween 80) was administered to conscious guinea pigs by oral gavage, not earlier than 2 weeks after implantation of the telemetry system. First, the effect of single doses of 0 (vehicle, n = 8), 0.1, 0.3, 1, 3, 10 or 30 mg/kg (n = 4–6 per dose group) on heart rate and ECG intervals was determined.

To study desensitization, we measured in a first experiment the effect of repeated oral administration of 3 mg/kg ponesimod (n = 6) on heart rate and ECG. Ponesimod was given four times to each guinea pig, at time 0, 3, 6, and 9 h. In a second experiment, the time interval between the first and the second dose of ponesimod was varied. At time 0 h, conscious guinea pigs (n = 2–4) were treated with the first dose (1 mg/kg) of ponesimod. The second dose (1 mg/kg) was administered 12 h after the first dose (Group 1), 24 h after the first dose (Group 2), 36 h after the first dose (Group 3) or 48 h after the first dose (Group 4).

Finally, we tested in a third experiment an up-titration regimen, in which eight doses of ponesimod were administered at 12-h intervals: 4 doses of 0.1 mg/kg, three doses of 0.2 mg/kg, and one dose of 0.4 mg/kg (n = 4). The effects of a 0.4 mg/kg dose given after up-titration were compared with the effects of the same dose without the up-titration (n = 4).

### Measurement of Plasma Ponesimod Concentration

At different time points after oral administration of ponesimod to satellite, non-implanted guinea pigs (n = 3–4 per time point), a blood sample was collected (5% EDTA); plasma was prepared and stored at −20°C. The plasma samples were analyzed for ponesimod concentration using liquid chromatography coupled to mass spectrometry (LC-MS/MS).

### Measurement of Blood Lymphocyte Count

Non-implanted male Dunkin Hartley guinea pigs (Harlan Laboratories, Horst, the Netherlands) were orally administered with ponesimod (10 mg/kg) or its vehicle (1 ml/kg). Three and 6 h after oral administration, guinea pigs were anesthetized by inhalation of 2–3% isoflurane and blood samples were collected retro-orbitally. For the determination of blood lymphocyte count, undiluted blood was analyzed using a Beckman Coulter 5diffCP hematology analyzer (Beckman Coulter, Zürich, Switzerland).

### Test Compound

Ponesimod (ACT-128800, Actelion Pharmaceuticals Ltd, Allschwil, Switzerland) was stored at room temperature (max. 25°C) protected from light. For oral administration, ponesimod was dispersed in methylcellulose 0.25% containing 0.05% (v/v) Tween 80.

### Statistical Analysis

Results, unless otherwise noted, are expressed as arithmetic mean ± standard error (SEM). Meaningful statistical analyses could not be performed because of the low number of animals used per group (n = 4 to 6).

## Results

### Effect of Single Oral Doses of Ponesimod on Heart Rate and ECG

**Figure 1:**
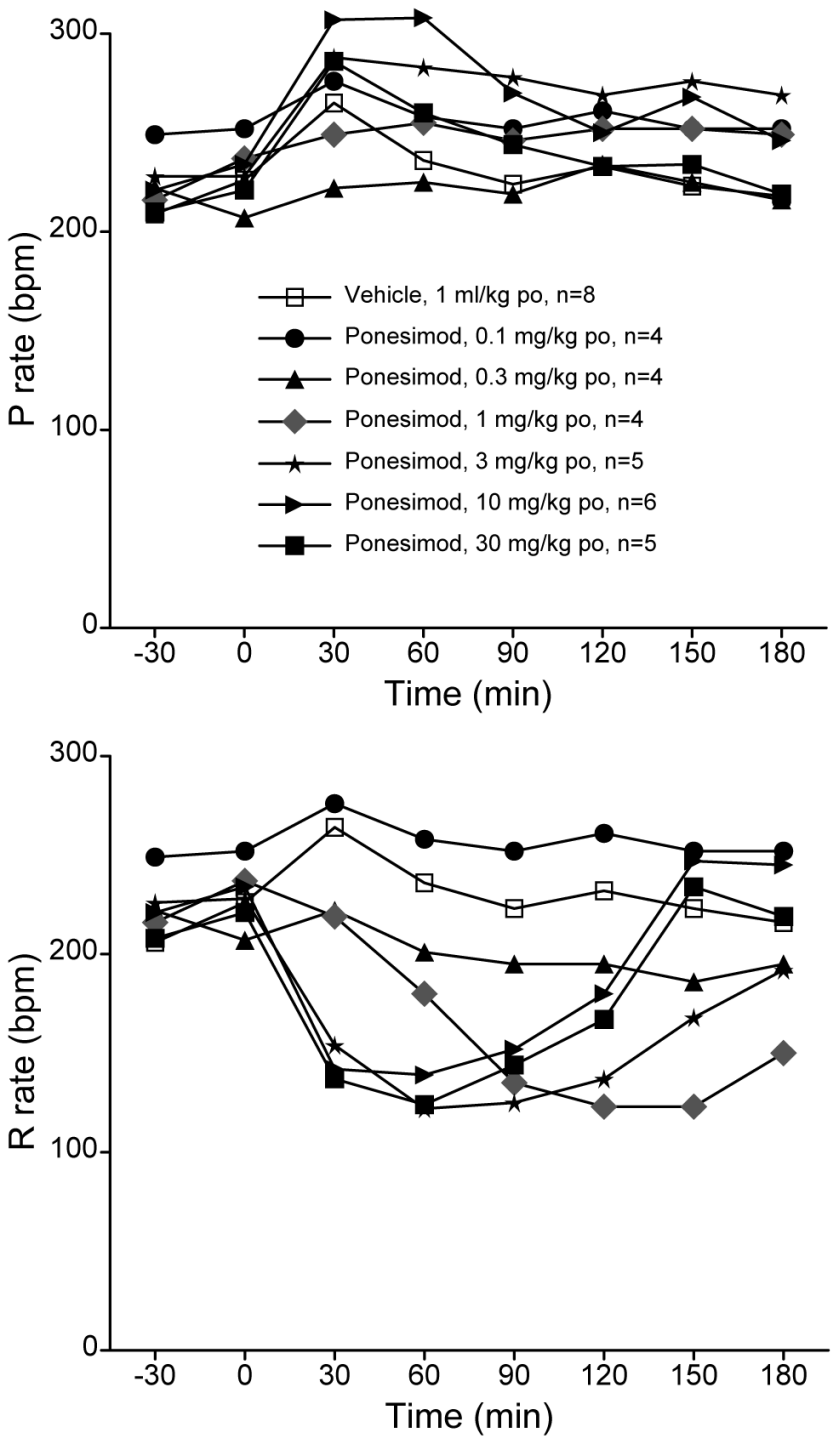
Effect of single oral doses of ponesimod on P and R rate in guinea pigs. Upper panel: P rate (P wave-to-P wave interval, i.e., sinus rate). Lower panel: R rate (R wave-to-R wave interval). Ponesimod (0.1, 0.3, 1, 3, 10 and 30 mg/kg) or vehicle (1 ml/kg) was administered by oral gavage to conscious guinea pigs implanted with telemetry transmitters. ECG data were collected continuously, over 10 s at 1-min intervals. Data are presented as means.

**Figure 2:**
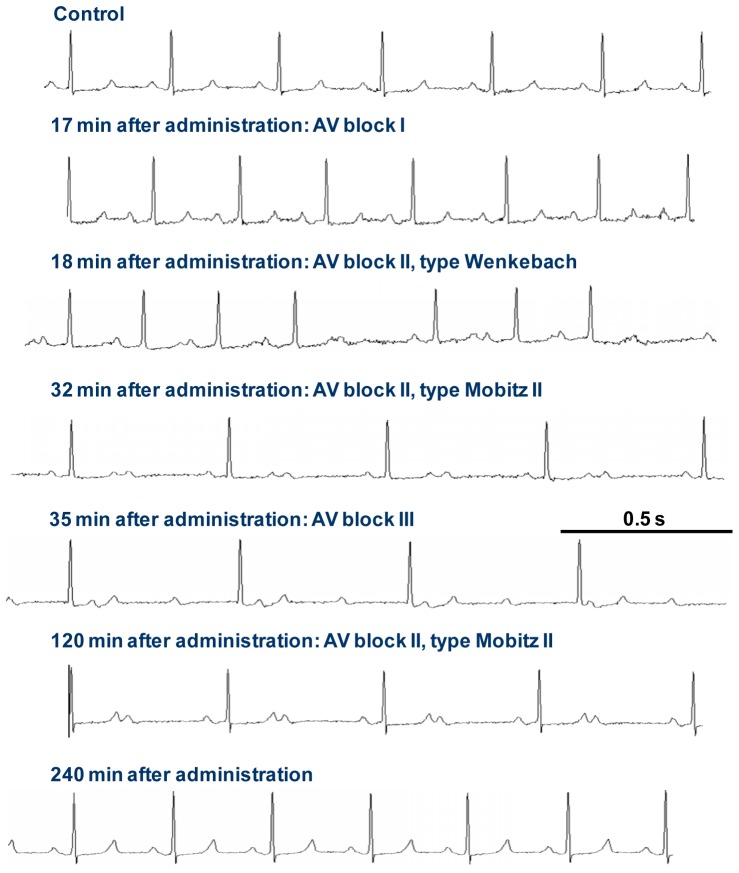
Effect of a single oral dose of ponesimod (3 mg/kg) on ECG waveforms. Representative ECG tracings from a conscious guinea pig implanted with a telemetry transmitter taken at baseline and 17, 18, 32, 35, 120, and 240 min after oral administration are shown.

Oral administration of ponesimod to conscious, freely moving guinea pigs implanted with a telemetry device resulted in a dose-dependent decrease in heart rate and changes in ECG intervals. Analysis of the ECG tracings of ponesimod-treated guinea pigs showed no dose-dependent effect on the sinus rate (i.e., the P wave-to-P wave interval), indicating that ponesimod did not induce sinus bradycardia (Figure 1, upper panel). However, single oral doses of 0.3 to 30 mg/kg of ponesimod transiently decreased the ventricular rate (R rate) in a dose-dependent manner (Figure 1, lower panel). An R rate decrease of 40% - as observed at doses of 1 mg/kg ponesimod or greater - is indicative of AV block type II, Mobitz II or AV block type III. In general, the following sequence of ECG changes was observed: first, the PR interval increased (indicative of AV block I), then QRS complexes started to disappear (indicative of AV block II, Wenckebach) until a 2∶1 conduction was observed (indicative of AV block II, Mobitz II) and later, a complete dissociation between P waves and QRS complexes was seen (indicative of AV block III). Representative ECG tracings from a guinea pig treated with 3 mg/kg ponesimod are shown in Figure 2. All effects on ECG variables had resolved 240 min after oral administration (Figure 2). A dose-dependent decrease of mean arterial pressure was observed following single oral administrations of ponesimod and appeared to be a consequence of the AV conduction delays (data not shown).

The dose dependence of the effect of ponesimod on ECG variables is summarized in Table 1. At a dose of 0.1 mg/kg, ponesimod did not induce any delay or interruption of the conduction between atrium and ventricle; i.e., no AV block I, II and/or III was observed. At a dose of 0.3 mg/kg, in four out of four treated guinea pigs the PR interval increased (AV block I). Two of the four also showed AV block II and in one guinea pig a complete dissociation between P waves and QRS complexes (AV block III) was observed. At single oral doses of 3 mg/kg or higher, ponesimod induced AV block I, II and III in all four to six treated guinea pigs (Table 1).

**Table 1:** Percent of guinea pigs showing AV block type I to III after a single oral dose of ponesimod.

	Dose ponesimod *mg/kg*						
	0(Vehicle)	0.1	0.3	1	3	10	30
	n = 6	n = 4	n = 4	n = 4	n = 4	n = 6	n = 6
AV block I	0	0	100	100	100	100	100
AV block II	0	0	50	100	100	100	100
AV block III	0	0	25	75	100	100	100

### Desensitization upon Repeated Oral Dosing

**Figure 3:**
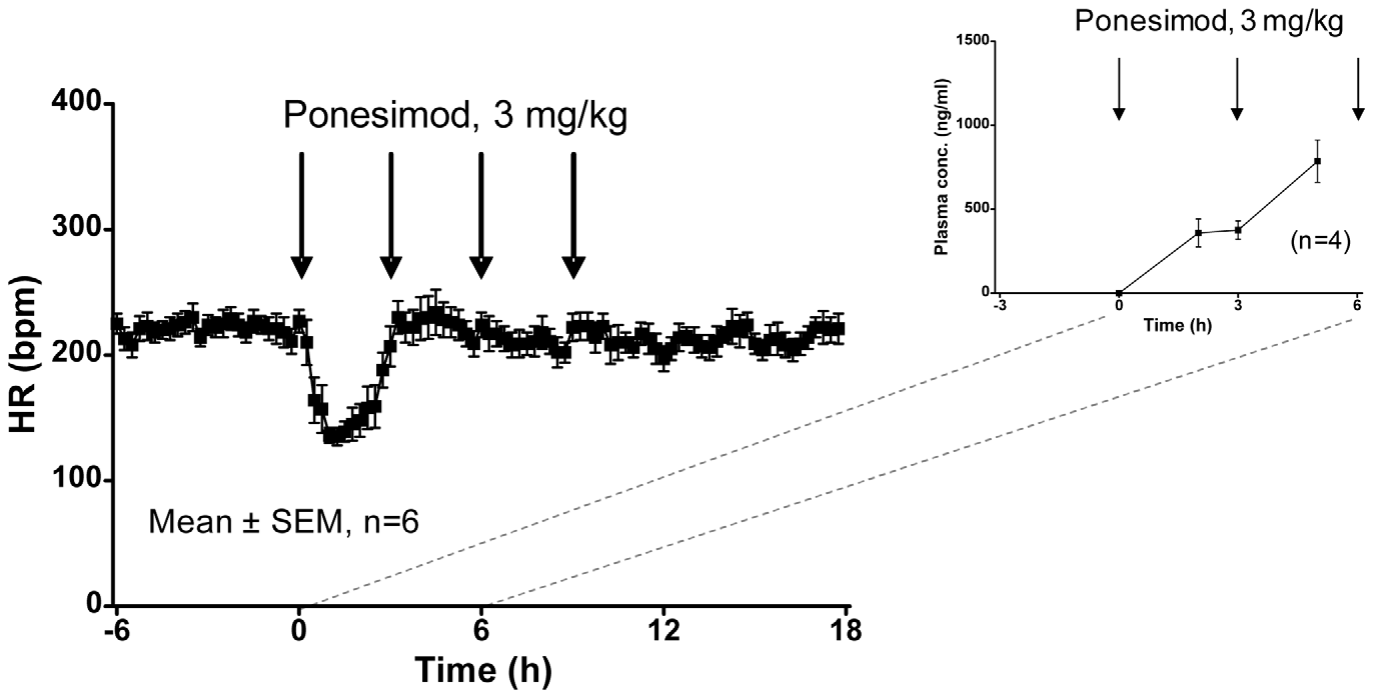
Effect of repeated oral administration of ponesimod (3 mg/kg) on heart rate. Heart rate (HR) was derived from telemetric blood pressure recordings in conscious guinea pigs. Data are presented as means ± SEM. Inset: Plasma concentration of ponesimod following the first two oral administrations of 3 mg/kg, dosed at 0 h and 3 h, in satellite animals. Data are presented as means ± SEM.

After having established a dose-effect relationship with single oral doses of ponesimod (Figure 1, Table 1), we investigated the effect of repeated administrations on ECG intervals. Ponesimod (3 mg/kg) was given orally to six conscious guinea pigs. Ponesimod was given four times to each animal, at 3-h intervals. Ninety minutes after the first administration of ponesimod, heart rate decreased by 36±3% (Figure 3). Heart rate returned to pre-dose values within 3 h. After the second, the third, and the fourth administration of 3 mg/kg ponesimod, no decrease in heart rate was observed anymore (Figure 3), indicating that the first dose of ponesimod had desensitized the system.

Analysis of the ECG tracings showed that during the first 3 h after the first oral dose of ponesimod AV block I, II and III occurred in each of the six treated guinea pigs. In contrast, no AV block II, Mobitz II or AV block III was observed after the second, the third, and the fourth administration of 3 mg/kg ponesimod in any of the guinea pigs. The plasma concentrations of ponesimod, measured in satellite animals, were 359±83 ng/ml at 2 h and 376±55 ng/ml at 3 h before administration of the second dose (Figure 3, inset). Two hours after the second administration of 3 mg/kg ponesimod (i.e. 5 h after the first dose) the plasma concentration reached 786±126 ng/ml. Thus, the second dose did not induce AV block II or III, indicating that despite a doubling of the ponesimod plasma concentration (at 5 h vs. 2 h time point), the system remained desensitized.

### Duration of the Desensitization Period

Next, we investigated how long the system remained desensitized after the first dose of ponesimod. For this purpose, a dose of 1 mg/kg, the lowest oral dose inducing AV block II, Mobitz II in all treated guinea pigs (see Table 1), was selected. At 0 h, guinea pigs were treated with the first oral dose (1 mg/kg) of ponesimod. The second dose (1 mg/kg) was administered 12 h later (Group 1), 24 h later (Group 2), 36 h later (Group 3) or 48 h later (Group 4). The effects on ECG variables are summarized in Table 2. The first dose induced AV block II, Wenckebach and AV block II, Mobitz II in all 12 treated guinea pigs (100%) and AV block III in 75% of the guinea pigs. When the second dose was administered 12 h after the first dose (Group 1), no AV block type II or III was observed and only 25% of the guinea pigs showed an AV block I. When the second dose was administered 24 h (Group 2) or 36 h (Group 3) after the first dose, 33% of the guinea pigs in each group showed AV block II, Mobitz II. When the second dose was administered 48 h after the first dose (Group 4), AV block II, Mobitz II was observed in 100% of the guinea pigs.

**Table 2:** Effect of dosing interval on incidence of AV block.

	1^st^ dose	2^nd^ dose	2^nd^ dose	2^nd^ dose	2^nd^ dose
	0 h	12 h	24 h	36 h	48 h
	(n = 12)	(n = 4)	(n = 3)	(n = 3)	(n = 2)
		Group 1	Group 2	Group 3	Group 4
AV block I, *%*	100	25	33	100	100
AV block II Wenckebach, *%*	100	0	33	33	100
AV block II Mobitz II, *%*	100	0	33	33	100
AV block III, *%*	75	0	0	0	0
Plasma concentration^a^	na	75±5	28±2	17±3	9±1

Two doses of ponesimod, 1 mg/kg each, were administered orally to conscious guinea pigs. Plasma concentrations were measured before the second dose, in satellite animals.

a ng/ml (mean ± S.D.), determined in satellite, non-implanted guinea pigs (n = 3–4 per time point).

na = not applicable.

Resensitization of the cardiac system was correlated with the plasma concentration of ponesimod: the lower the plasma concentration at the time of the second administration, the more pronounced the effect of the second dose on the ECG variables. No AV block II or III was observed when the second dose was given 12 h after the first dose. Once the plasma concentration dropped to 9±1 ng/ml (48 h after the first dose), the system had re-established its high sensitivity towards S1P_1_ receptor agonism (Table 2).

### Desensitization by Using an Up-titration Regimen

Finally, it was investigated whether the cardiac system in the guinea pig could be desensitized without a first-dose effect on the ECG by using an up-titration regimen. For these experiments, an oral dose of 0.1 mg/kg was selected as the starting dose because this dose of ponesimod did not induce any AV blocks (Table 1). Additional doses were then administered with the aim to finally administer a dose of ponesimod that, when given for the first time, induces AV blocks II in all guinea pigs.

In pilot experiments the frequency of administration of 0.1 mg/kg ponesimod was varied. When the time interval between the first and second dose was 1 h, AV blocks II were induced by the second dose in the three treated guinea pigs. In contrast, when the time interval between doses was 3 h, no AV block II or III was observed after the second and third dose, suggesting that the increase in plasma concentration, due to the 1 h time interval, was faster than the desensitization process induced by the first dose.

**Figure 4:**
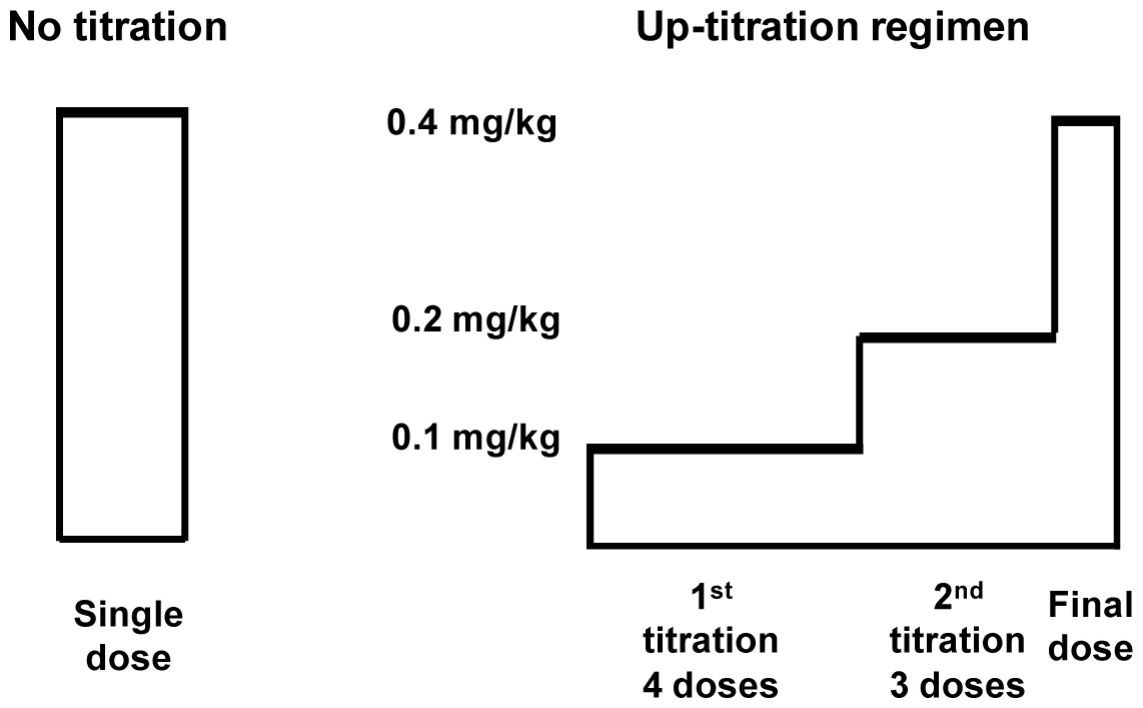
Design of up-titration regimen.

Based on these data, the following up-titration experiment was performed: Four conscious guinea pigs were first treated with four consecutive doses of 0.1 mg/kg ponesimod, followed by three doses of 0.2 mg/kg and finally with one dose of 0.4 mg/kg (Fig. 4). The eight doses were administered using a dosing interval of 12 h, corresponding to the half-life of ponesimod in the guinea pig. For comparison, four conscious guinea pigs were treated once with 0.4 mg/kg ponesimod. The ECG of each guinea pig was continuously monitored, the ECG tracings were analyzed as described, and the data are presented in Table 3. A single oral administration of 0.4 mg/kg ponesimod induced AV block I and AV block II, Wenckebach in all four treated animals (100%). Furthermore, in three out of four guinea pigs (75%) AV block type II, Mobitz II was observed. Using an up-titration regimen, no AV block II was observed, and only two of the four treated guinea pigs (50%) showed an AV block I (PR interval prolongation), indicating that ponesimod can desensitize the cardiac system in the absence of a significant first-dose effect.

**Table 3:** Incidence of AV block after a single oral dose of 0.4/kg ponesimod given as a first dose or after an uptitration regimen.

	No up-titration	Up-titration
	*0.4 mg/kg*	*0.1–0.2–0.4 mg/kg*
	n = 4	n = 4
AV block I, *%*	100	50
AV block II Wenckebach, *%*	100	0
AV block II Mobitz II, *%*	75	0
AV block III, *%*	0	0

Up-titration regimen: 4×0.1 mg/kg, 3×0.2 mg/kg, 1×0.4 mg/kg, 12 h between doses.

## Discussion

S1P and the S1P_1_ receptor are essential for lymphocyte trafficking in all species studied and regulate egress of T and B cells from thymus and peripheral lymphoid organs [5], [6]. Selective S1P_1_ modulators, like ponesimod, reduce peripheral lymphocyte count, and are under investigation for treatment of lymphocyte-mediated autoimmune diseases [14], [15]. In humans, the S1P_1_ receptor is also involved in the regulation of heart rate and rhythm [7], [16]. This is in contrast to mice and rats, in which S1P_1_ modulators that do not activate the S1P_3_ receptor are not lowering heart rate following oral dosing [8], [9], [17].

In the present study, conscious guinea pigs equipped with a telemetry device were used for the electrophysiological evaluation of the effects of ponesimod on heart rate and AV conduction. In this species, ponesimod compared to vehicle treatment reduced the blood lymphocyte count by 60% and 68%, at 3 h and 6 h after a single oral dose of 10 mg/kg. In addition, and in contrast to rats and dogs, ponesimod dose-dependently reduced heart rate in guinea pigs by inducing AV conduction delays. The cardiac system became rapidly desensitized by ponesimod, and understanding of the desensitization and resensitization kinetics in relation to the plasma concentration of ponesimod in guinea pigs allowed us to design an up-titration regimen resulting in significant reduction of AV conduction delays. This knowledge was then translated to a dosing regimen of ponesimod to be tested in humans to prevent or significantly reduce the first-dose effects on heart rate and AV conduction.

Desensitization is a well-known adaptation phenomenon, described for a number of agonistic principles [18.19]. For GPCRs, agonist stimulation leads to G protein coupling and activation, followed by receptor phosphorylation. Phosphorylated receptors recruit cytosolic β-arrestins; their binding prevents further G protein coupling and, therefore, leads to desensitization of G protein-dependent signaling. The receptor-bound β-arrestins facilitate receptor internalization and the internalized receptors either recycle to the cell surface or are degraded [20]. Resensitization to agonist stimulation, therefore, requires either receptor recycling or receptor neosynthesis. In cellular systems, it was shown that ponesimod causes efficient receptor internalization, degradation, and functional antagonism [21].

The guinea pig model allowed us to study *in vivo* the different aspects of ponesimod-induced receptor activation, receptor desensitization, and resensitization of the S1P_1_ system in the heart.

First, ponesimod dose-dependently reduced heart rate by causing AV conduction delays. The dose of the S1P_1_ receptor agonist required to induce at least AV block type I in all treated animals (4 out of 4) was low (0.3 mg/kg p.o.), corresponding to a plasma concentration at onset of approximately 10 ng/ml as measured in satellite guinea pigs. A single oral dose of 3 mg/kg induced AV block type I, II and III in all treated animals. The mechanisms underlying the changes in heart rate and rhythm upon activation of S1P receptors have not been fully elucidated and appear to be different between species. In a mouse study [22], it was shown that the non-selective S1P modulator fingolimod dose-dependently reduced heart rate in wild-type mice, whereas in GIRK 4^−/−^ knockout mice lacking the G protein-gated inwardly rectifying potassium channel (Kir3.1/3.4), no heart rate decrease was observed. Furthermore, the sustained reduction of heart rate induced by fingolimod in wild-type mice was also abolished in S1P_3_^−/−^ knockout mice [9]. These data suggest that in mice, binding of fingolimod to the S1P_3_ receptor leads to activation of the muscarinic potassium current, *I*_KACh_, probably via direct interaction of GIRK with the βγ dimer formed by dissociation of the activated G protein [16], [23]. Consistent with this mode of action is the fact that a selective S1P_1_ agonist had no effect on heart rate in wild-type mice [9]. Studies using freshly isolated guinea pig and mouse atrial cardiomyocytes revealed clear species differences in the activation of the muscarinic potassium current, *I*_KACh_ [24], [25], [26]. The S1P receptor agonist sphingosylphosphorylcholine (SPPC), for example, potently activated *I*_KACh_ in guinea pig cardiomyocytes but not in mouse cardiomyocytes [25].

Second, desensitization of the cardiac system occurred rapidly in ponesimod-treated guinea pigs and could be maintained by repeated dosing. One hour after administration of a single oral dose of 3 mg/kg ponesimod, the heart rate was maximally (i.e. about 50%) decreased and the ECG showed AV block II, Mobitz and AV block III. Three hours after the 1^st^ dose, the heart rate was back to pre-dose values, even though the plasma concentration was higher at 3 h than at 2 h after dosing. Repeated administration of 3 mg/kg ponesimod maintained desensitization, suggesting that despite increasing concentrations of ponesimod, the initially observed G protein-dependent signaling no longer occurred. When the time interval between the doses was shortened, from 3 h to 1 h, repetitive dosing of ponesimod caused heart rate changes, indicating that the rapid increase of plasma concentration triggered G protein-dependent signaling. Thus, desensitization was time dependent, probably because β-arrestin recruitment followed by receptor internalization is a slower process than G protein activation.

Third, resensitization of the S1P_1_ system in the heart is dependent on the S1P_1_ receptor agonist concentration. After desensitization had been induced by a first dose of ponesimod, the cardiac system remained desensitized as long as the plasma concentration was ≥75 ng/ml. When the concentration decreased due to drug clearance, the system started to become resensitized and reached full sensitivity again at a plasma concentration of 9 ng/ml or below. In the time period of decreasing plasma concentration (i.e., from 75 to 9 ng/ml), presumably more and more functional S1P_1_ receptors became present on the surface of cardiomyocytes as the result of either receptor recycling or neosynthesis.

Fourth, the effects of ponesimod in guinea pig did not completely mimic the effects in humans: we did not observe sinus bradycardia, which has been seen in clinical studies with ponesimod [7] and is a known effect of fingolimod [10]. In ponesimod-treated humans, heart rate reductions due to sinus bradycardia are seen concomitantly with a reduction in the peripheral lymphocyte count. In guinea pigs, AV conduction delays occurred in each treated guinea pig, whereas in humans conduction delays were only occasionally observed. Both the sinus bradycardia and the delayed AV conductance are likely related to activation of GIRK (Kir3.1/3.4), mediated by S1P receptors in cardiomyocytes of the sinoatrial and atrioventricular nodes, respectively. The effects of acetylcholine and adenosine in isolated hearts suggest that AV conductance in the guinea pig may be particularly sensitive to GIRK activation [27], [28]. However, differences in resting parasympathetic tone, tissue distribution of receptors, distribution or activity of regulators of G proteins [29], or a combination of factors could account for the observed species differences in sensitivity to S1P_1_ receptor modulators.

Overall, our findings suggest that the first-dose effects of the selective S1P_1_ receptor modulator ponesimod on heart rate and AV conduction is due to activation of the S1P_1_ receptor in guinea pigs as well as in humans. In the presence of sufficient concentrations of ponesimod, this initial receptor activation is rapidly followed by desensitization of the S1P_1_ system in cardiomyocytes but also in lymphocytes, leading to normalization of heart rate and rhythm and prevention of lymphocyte egress from secondary lymphoid organs, respectively. Thus, functional antagonism by selective S1P_1_ receptor modulators such as ponesimod is a key mechanism potentially resulting in an efficacious and safe therapy of multiple sclerosis, psoriasis and other autoimmune diseases.
